# The insulin-like growth factor 2 mRNA-binding protein 2 affects tumor cell metabolism *via* mitochondrial transporter activity and lipid alterations

**DOI:** 10.1038/s41420-026-03315-4

**Published:** 2026-09-01

**Authors:** Sandra Kendzia, Susanne Franke, Marcello Pirritano, Eva Grafahrend-Belau, Nikolai Köhler, Theresa Bauchspiess, Sören Franzenburg, Marcus Höring, Elia Raab, Linus Schmeier, Sinem Sürmeli, Frank Erdmann, Johannes Klose, Jörg Kleeff, Michael Boettcher, Gerhard Liebisch, Josch K. Pauling, Martin Simon, Björn H. Junker, Sonja M. Kessler

**Affiliations:** 1 Experimental Pharmacology for Natural Sciences, Institute of Pharmacy, Martin Luther University Halle-Wittenberg, Halle (Saale), Germany; 2 Biosynthesis of Active Substances, Institute of Pharmacy, Martin Luther University Halle-Wittenberg, Halle (Saale), Germany; 3 Molecular Cell Biology and Microbiology, University of Wuppertal, Wuppertal, Germany; 4 LipiTUM, Chair of Experimental Bioinformatics, TUM School of Life Sciences, Technical University of Munich, Freising, Germany; 5 Competence Centre for Genomic Analysis, Kiel, Germany; 6 Institute for Clinical Chemistry and Laboratory Medicine, University of Regensburg, Regensburg, Germany; 7 Department of Visceral, Vascular and Endocrine Surgery, University Medical Center Halle (Saale), Martin Luther University Halle-Wittenberg, Halle (Saale), Germany; 8 Institute of Molecular Medicine, Martin Luther University Halle-Wittenberg, Halle (Saale), Germany; 9 Halle Research Centre for Drug Therapy (HRCDT), Halle (Saale), Germany; 10 CIOBio, Institute for Clinical Chemistry and Laboratory Medicine, University Hospital and Faculty of Medicine Carl Gustav Carus, Dresden University of Technology, Dresden, Germany

**Keywords:** Cancer metabolism, Mechanisms of disease

## Abstract

The insulin-like growth factor 2 mRNA-binding protein (IGF2BP) family is overexpressed in cancer and associated with poor prognosis. IGF2BP2 has been linked to single metabolic alterations by acting on its RNA targets. Here, we used a comprehensive approach to elucidate the effects of IGF2BP2 on primary and lipid metabolism. ^13^C-metabolic flux analysis (MFA) combined with RNA-Seq data revealed that IGF2BP2 affects mitochondrial fluxes by regulating the expression of several mitochondrial transporters, such as mitochondrial pyruvate carrier 1 (MPC1) and uncoupling protein 2 (UCP2). Methyl pyruvate reversed the gene expression patterns of *UCP2* and *CPT1A* in HCT116 *IGF2BP2* knockout (KO) cells by bypassing MPC1. Interestingly, an altered expression of the transporter UCP2 was also observed in a patient-derived tumor organoid (PDO), in which IGF2BP2 was knocked down. The altered glutamine metabolism seen in the ^13^C-MFA and the citrate label data derived from extracted mitochondria confirm a rerouting of glutamine almost exclusively into the mitochondria and a reduction of glycolytic carbon intake into the mitochondria. Due to changes in palmitate labeling patterns, lipid stainings were performed, suggesting lipid accumulation in KO cells. A lipidomic analysis revealed altered compositions across almost all lipid species. Further, lipogenic genes involved in fatty acid and cholesterol metabolism were differentially expressed. Most of the differentially expressed genes are potential direct targets of IGF2BP2 based on publicly available IGF2BP2 CLIP data. Overall, these results show the influence of IGF2BP2 on the central carbon metabolism of cancer cells, primarily through its effects on MPC1 and the resulting effects on UCP2. The complex interaction of IGF2BP2 with the metabolic network provides important insights into tumor metabolism, particularly relevant to tumor growth and resistance to therapy.

## Introduction

As an RNA-binding protein, the insulin-like growth factor 2 mRNA-binding protein 2 (IGF2BP2/IMP2/p62) promotes RNA stability, localization, modification, and processing [1–3]. As a result, IGF2BP2 is involved in a wide range of biological processes, including embryonic development, lipid metabolism, neuronal differentiation, insulin resistance, and tumorigenesis [4]. IGF2BP2 is overexpressed in several tumor entities as well as linked with poor patient survival in different cancer types [5–11]. Overexpression of IGF2BP2 leads to genomic instability and promotes cancer cell proliferation and migration [6, 12, 13]. Additionally, IGF2BP2 was shown to be a reader of N6-methyladenosine (m6A) [14], a posttranscriptional modification of RNA that regulates gene expression by altering RNA processing [15].

With regard to metabolism, IGF2BP2 has been described to affect glucose and lipid metabolism. IGF2BP2 regulates cellular metabolism across a variety of cell types and pathways through posttranscriptional regulation of multiple genes [1]. A liver-specific deletion of IGF2BP2 reduces hepatic fatty acid oxidation and increases hepatic triglyceride accumulation in mice [16]. Furthermore, *Igf2bp2−/−* mice are resistant to diet-induced obesity and fatty liver [17]. In contrast, a liver-specific overexpression of IGF2BP2 induces higher hepatocytic triglyceride storage in mice [18]. IGF2BP2 promotes fatty acid elongation in liver cancer cells and an alteration of lipogenic genes in human liver cancer tissue [19, 20]. In addition to lipid metabolism, individual studies linked IGF2BP2 expression to glucose metabolism. IGF2BP2 promotes glycolysis by binding to and stabilizing solute carrier family 2 member 1 (*GLUT1)* and hexokinase 2 (*HK2*) mRNA [21]. Similarly, IGF2BP2 promotes aerobic glycolysis by *GLUT1* stabilization, which leads to a poor prognosis in pancreatic ductal adenocarcinoma [22]. Furthermore, IGF2BP2 also stimulates tumor progression through Myc-mediated glycolysis [23].

In addition to glucose and lipid metabolism, there is emerging evidence of a possible link between IGF2BP2 and mitochondrial function, although further research is needed to fully elucidate the mechanisms underlying these observations. Janiszewska et al. [24] reported that IGF2BP2 regulates energy metabolism by inducing oxidative phosphorylation (OXPHOS) in glioblastoma cells. In the latter study, *IGF2BP2* depletion led to a decreased oxygen consumption rate (OCR) and respiratory complex I and IV activity. In mice, an *Igf2bp2* KO leads to increased energy expenditure without changes in mitochondrial oxidative capacity, but with increased mitochondrial DNA and reduced maximal oxygen consumption [17]. Due to these contradictory effects, the authors of the latter study speculated that IGF2BP2 might also be responsible for the transport and/or localization of mRNA into the mitochondria.

In summary, these studies have shown individual effects on various metabolic factors, but the overall relationship among these findings remains unclear. To elucidate the full picture of IGF2BP2-induced metabolic changes, we used a comprehensive approach: ^13^C-MFA combined with lipidomic and transcriptomic analyses of HCT116 *IGF2BP2* knockout (KO) cells. We confirmed the relevance of the main findings by rescue assays and experiments using a patient-derived organoid (PDO).

## Results

### Mitochondrial fluxes are decreased in *IGF2BP2* KO cells, while energy metabolism is maintained

**Fig. 1:**
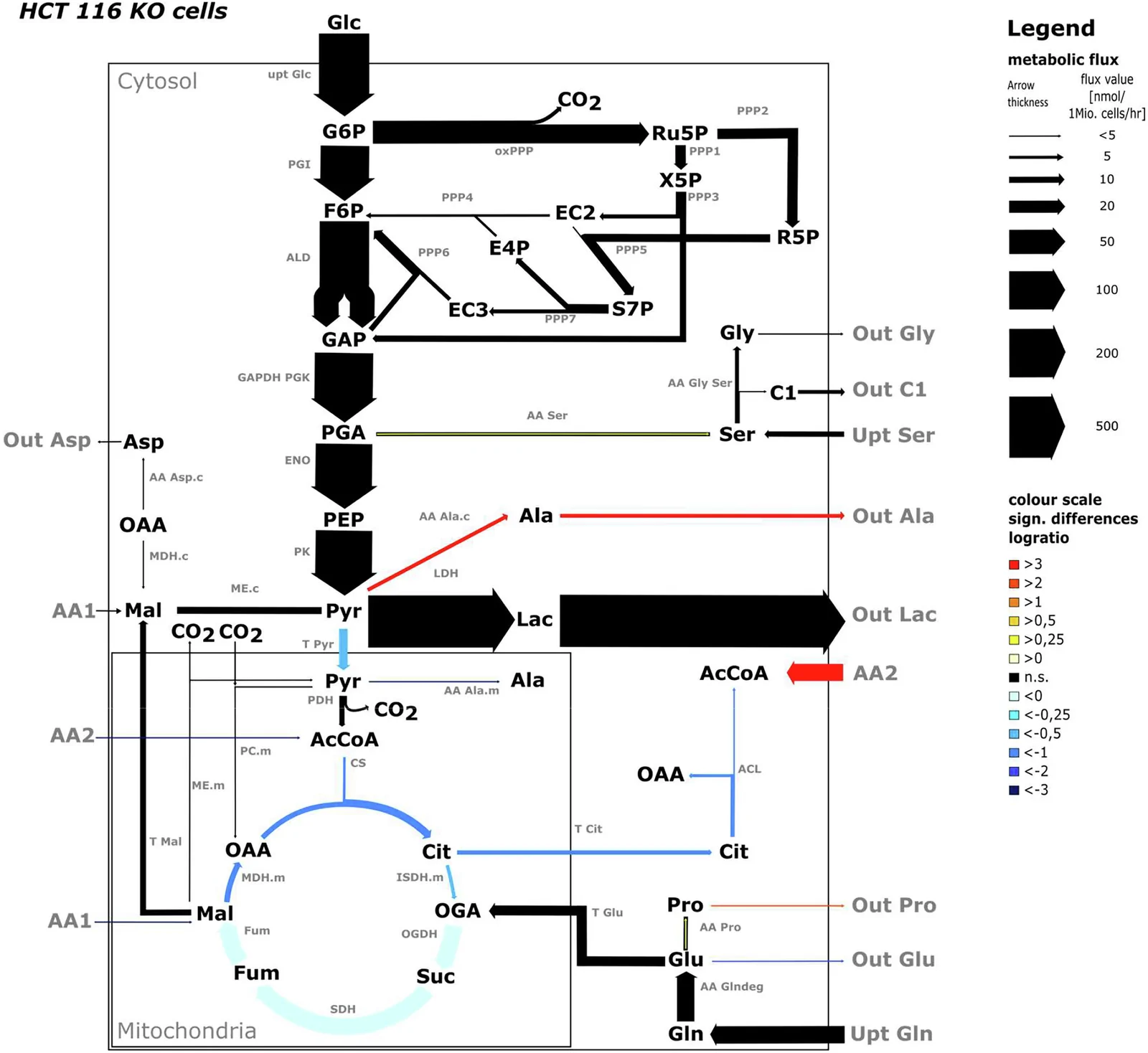
Mitochondrial fluxes are decreased in *IGF2BP2* knockout cells. Metabolic flux map of the central carbon metabolism of HCT116 IGF2BP2 knockout (KO) cells (for HCT116 wild type (WT) cells see Fig. S1A), including glycolysis, pentose phosphate pathway, TCA cycle, as well as anaplerotic reactions. The arrows depict the absolute flux values of the net carbon flux through the reaction; the direction and thickness of the arrow depict the absolute flux value and net direction. Significantly changed fluxes in KO cells compared to WT cells are colored according to the logratio scale. Red indicates increased flux; blue indicates decreased flux. The reaction IDs are shown next to the arrows, for abbreviations and corresponding reactions see Table S1. For absolute flux values [nmol/10^6cells/h] see Fig. S1. *n* quadruplicate.

To investigate the full spectrum of metabolic changes induced by IGF2BP2 in colon cancer cells, a ^13^C-metabolic flux analysis was performed using Isotopomer Network Compartmental Analysis (INCA) [25, 26]. The flux map of the HCT116 wild-type (WT) cells revealed typical cancer metabolism, namely aerobic glycolysis and a reduced tricarboxylic acid cycle (TCA) (Fig. S1A) [27, 28]. The estimated fluxes of the ^13^C-MFA, as well as the external rates and labeling data, are listed in Table S1. In HCT116 KO cells, almost all calculated mitochondrial fluxes were significantly reduced compared to the WT fluxes (Fig. 1). The calculated transport reactions into the mitochondria T_Pyr, T_Cit, AA1 and AA2 (for reaction IDs and corresponding reactions, see Table S1) were significantly reduced (Fig. 1). The mitochondrial alanine production was reduced as well, whereas the cytosolic alanine production was increased (Fig. 1). The transport reaction T_Mal was inverted, the transport reaction T_Glu showed no difference (Fig. 1). The catabolic reaction from citrate to acetyl-CoA was significantly reduced, while the uptake of acetyl-CoA from the extracellular compartment into the cytosol significantly increased (Fig. 1). Furthermore, glutamine metabolism was significantly altered: the glutamate output was significantly decreased, whereas proline production and output were significantly enhanced (Fig. 1).

Additionally, a reduction in growth of the KO cells in comparison to the WT cells could be observed in the experimental setting of the ^13^C-MFA (see Table S1). This was reflected in a tendency of 12.5% reduction in glucose uptake and a 40.9% reduction in glutamine uptake of the KO cells in comparison to the WT cells. Furthermore, the uptake of other amino acids was enhanced (see Table S1).

**Fig. 2:**
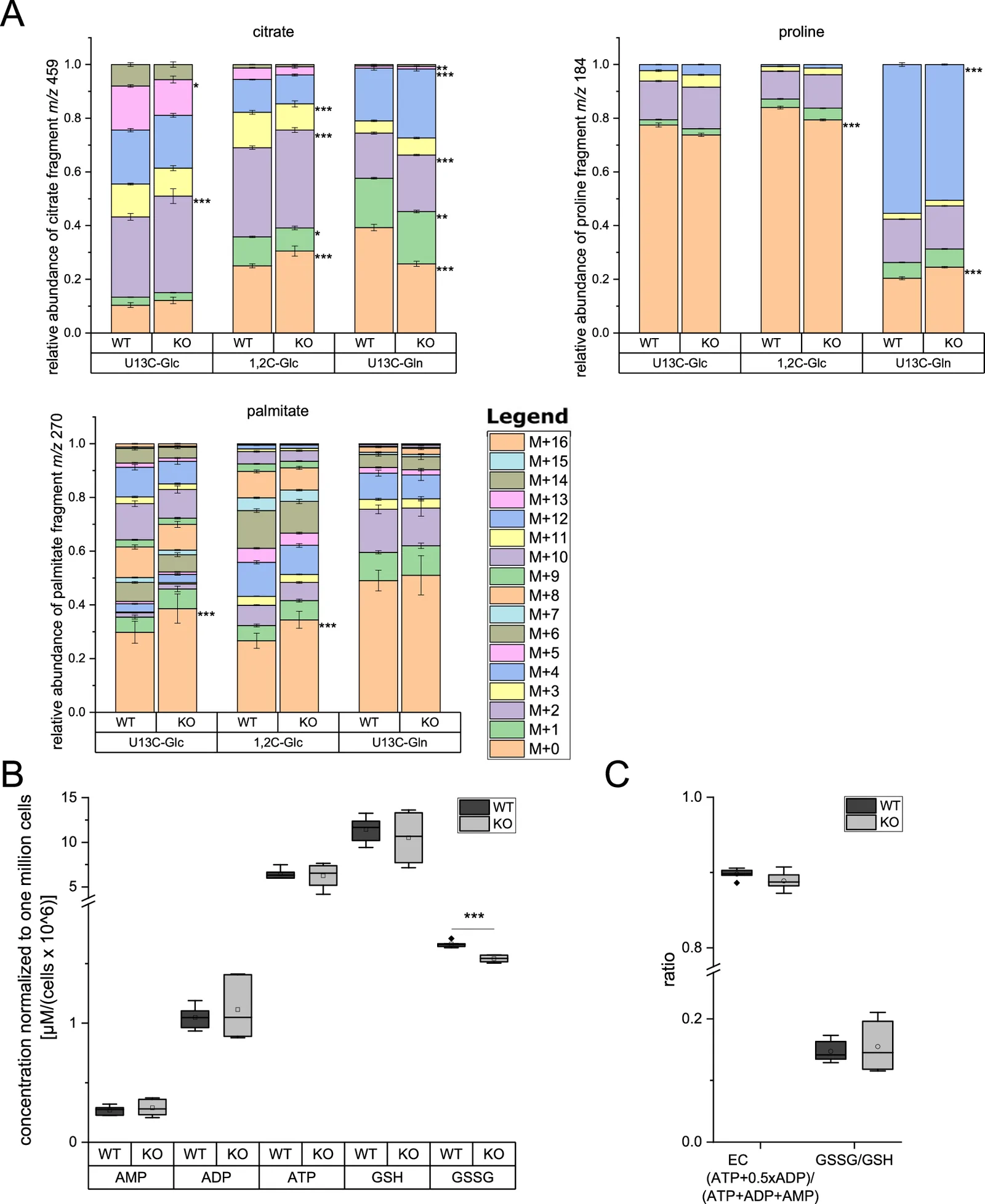
Mass isotopomer distributions confirm reduced mitochondrial fluxes while maintaining energy homeostasis in *IGF2BP2* KO cells. **A** Graphs of mass isotopomer distribution of fragments of proline, citrate and palmitate derived from U-^13^C-glucose (Glc), 1,2-^13^C-glucose and U-^13^C-glutamine (Gln) in HCT116 wild type (WT) cells and HCT116 IGF2BP2 knockout (KO) cells. Statistical significance was tested by two-way ANOVA, followed by a Tukey´s post hoc test. Data are shown as mean ± SD; *n* = quadruplicate. **B**, **C** After methanol/chloroform extraction of the control cells of the isotopic tracer experiment, the polar phases were used to determine the glutathione, AMP, ADP and ATP content in the WT and KO cells *via* LC/MS (**B**). The energy charge of the adenylate system (EC) was calculated, and the ratio of GSSG/GSH was determined (**C**). Statistical analysis was performed using Student’s *t* test as the data were normally distributed. Data are shown as box plots. The box is determined by the 25th and 75th percentiles, the whiskers by the 5th and 95th percentiles. Additionally included are the median (line), mean (non-filled symbol) and outliers (filled symbols). *n* = sextuplicate; *p ≤ 0.05, **p ≤ 0.01, ***p ≤ 0.001.

The corresponding mass isotopomer distributions (MIDs) of citrate, proline, and palmitate are shown in Fig. 2A, additional labeling data can be found in Table S1. In Fig. 2A, mass isotopomers are characterized as M + n, where n equals the number of labeled carbon atoms in the fragment. The MID from citrate and malate showed less label incorporation *via* the glucose tracers and considerably more label with U^13^C-glutamine (Fig. 2A and Table S1). The MIDs of aspartate, proline, and glutamate all show more label incorporation by the glucose tracers and less by the glutamine tracer in KO compared to WT (Fig. 2A and Table S1). No difference in label distribution between KO and WT cells was observed for the MID of palmitate using the U^13^C-glutamine tracer (Fig. 2A). With both glucose tracers, there was less label incorporated into palmitate in the KO compared to the WT (Fig. 2A).

Regarding energy metabolism, no difference in the measured ATP, ADP and AMP concentrations or in the glutathione/glutathione disulfide (GSH/GSSG) ratio was observed in the KO cells in comparison to the WT cells (Fig. 2B, C). The changed exchange rates, combined with consistent but slower growth of KO cells compared with WT cells, suggested a stable metabolic rewiring. Similarly, the unchanged GSH/GSSG ratio between the cell lines provided no evidence of a cellular stress response after *IGF2BP2* KO [29].

### Distinct changes in the lipidome of *IGF2BP2* KO cells point to fatty acid uptake, saturation, and elongation effects

**Fig. 3:**
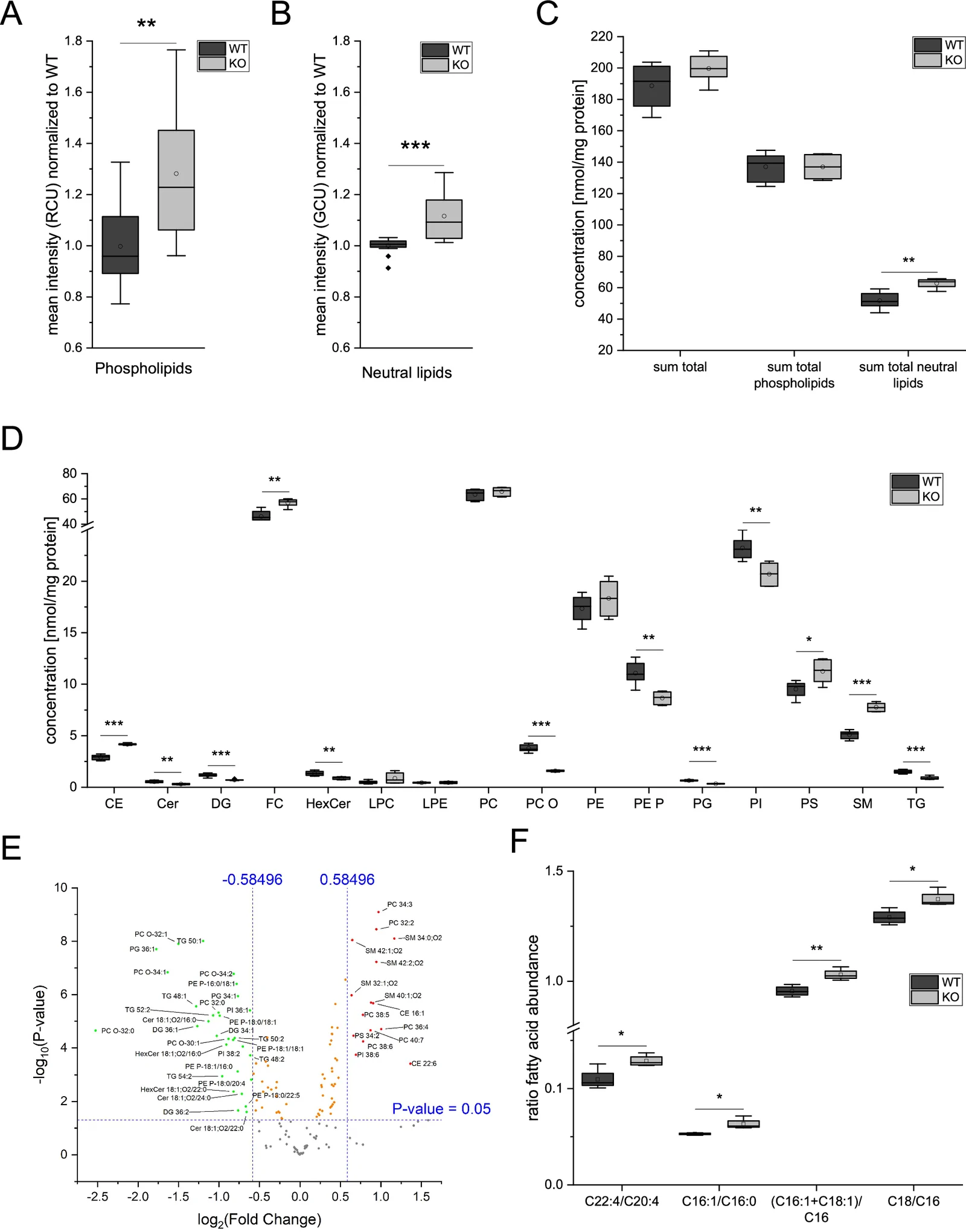
Lipidomic changes in *IGF2BP2* KO cells suggest increased fatty acid uptake. **A**, **B** Quantification of the mean fluorescence intensity after staining HCT116 wild type (WT) cells and HCT116 *IGF2BP2* knockout (KO) cells with HCS LipidTOX™ Red phospholipid detection reagent (**A**) and HCS LipidTOX™ Green neutral lipid stain (**B**). Fluorescence intensity was measured using the Incucyte® S3 system. Statistical analysis was performed using Student's *t* test (**A**) and Mann–Whitney *U* test (**B**) depending on the normal distribution of the data. *n* = 3 (quintuplicates). **C**, **D** Concentration of different lipid classes normalized to the protein content of the cells. Statistical analysis was performed using Student's *t* test or Mann–Whitney *U* test based on the normal distribution of the data. *n* = 2 (triplicate). CE cholesteryl ester, Cer ceramide, DG diglyceride, FC free cholesterol, Hex Cer hexosylceramide, LPC lysophosphatidylcholine, LPE lysophosphatidylethanolamine, PC phosphatidylcholine, PC O phosphatidylcholine-ether, PE phosphatidylethanolamine, PE P based plasmalogens, PG phosphatidylglycerol, PI phosphatidylinositol, PS phosphatidylserine, SM sphingomyelin, TG triglyceride. **E** Volcano plot of the fold changes (FC) of specific lipid species between HCT 116 *IGF2BP2* KO vs. HCT116 WT cells. All specific lipid species with an FC of at least ±1.5 and a *p* value under 0.05 were annotated. **F** Graphical depiction of significantly altered ratios of fatty acid amounts of WT and KO cells. Statistical analysis was performed using Student's *t* test. *n* = quadruplicate; *p ≤ 0.05, **p ≤ 0.01, ***p ≤ 0.001. Data shown as box plots (**A**–**F**): the box is determined by the 25th and 75th percentiles, the whiskers by the 5th and 95th percentiles. Additionally included are the median (line), mean (non-filled symbol) and outliers (filled symbols).

Due to the observed changes in the labeling pattern of palmitate in the isotopic labeling experiment, we stained the HCT116 WT and *IGF2BP2* KO cells with fluorescent dyes for neutral lipids and phospholipids. Both staining intensities were significantly higher in the KO cell line (Fig. 3A, B). To gain detailed information of lipid species of both cell lines a lipidomic analysis was conducted. The lipidomics data revealed altered compositions across almost all lipid species (Fig. 3D). By summarizing the different lipid species, the lipidomics data reflected the staining differences between the two cell lines for neutral lipids. For the total phospholipid content, no differences between the cells were observed (Fig. 3C).

The lipidomic data (full dataset, see Table S2) were then integrated into the lipid network explorer (Linex^2^) [30, 31], a web tool to analyze lipid metabolic networks. It generated a dataset-specific lipid interaction network and statistical data. Principal component analysis of the most relevant substructures clearly separated WT and KO cells based on the lipidomic data (Fig. S2). The visualized lipidomic network (Supplemental File S1) revealed significant alterations in the acetyltransferase reaction between phosphatidylcholine (PC; 34:1) and lysophosphatidylcholine (LPC; 18:1, 16:1).

The shift in palmitate labeling and changes in the C16- and C18-containing lipids PC and LPC led to the theory that fatty acid metabolism might be further affected by IGF2BP2. Therefore, the fatty acid compositions of WT and KO cells were measured by GC-MS (see Table S2). To determine not only changes in the fatty acid profiles but also to relate these to estimated enzyme activities, the measured amounts of saturated and unsaturated fatty acids were compared according to Hofmanova et al. [32]. Significant changes in the ratios of C22:4/C20:4, C16:1/C16:0, and C16:1 + C18:1/C16:0 corresponding to the estimated activities of the enzymes elongation of very long-chain fatty acids protein 5/2 (ELOVL 5/2) and stearoyl-CoA desaturase (SCD) were observed (Fig. 3F). The ratio of the sum species C18 to the sum species C16 was significantly higher in the KO vs. WT cells, reflecting the estimated activity of the enzyme ELOVL6 [33, 34] (Fig. 3F).

### IGF2BP2 affects gene expression of both mitochondrial transporters and lipogenic factors

In addition to the altered metabolic phenotype, transcriptional alterations were observed in IGF2BP2 KO cells compared to WT cells. RNA-Seq data (see Table S3) of both cell lines, followed by an enrichment analysis, highlighted that genes with decreased expression were involved in OXPHOS (second hit on the list; Fig. S3).

**Fig. 4:**
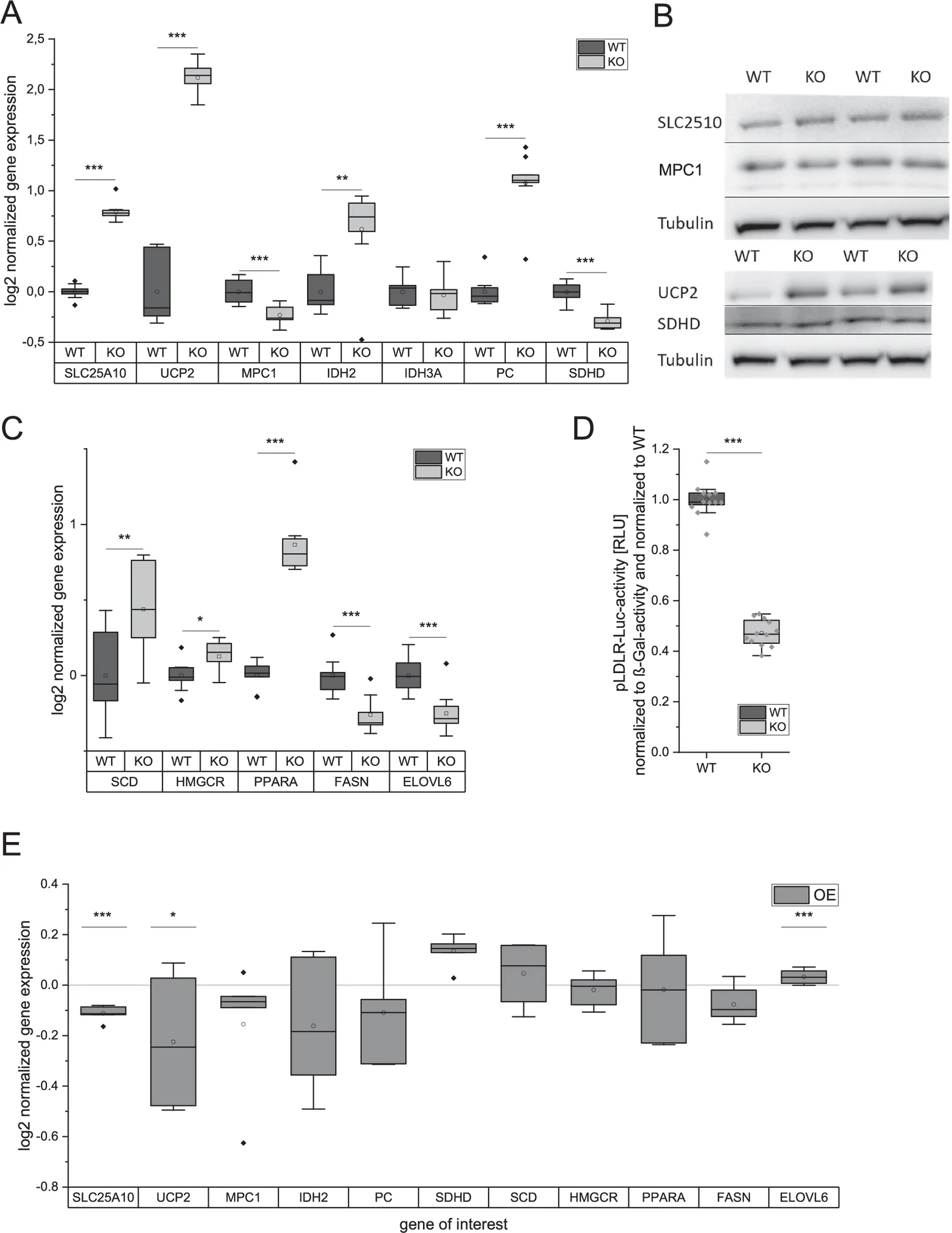
IGF2BP2 affects gene expression of both mitochondrial transporters and lipogenic factors. **A**, **C** Gene expression of genes of the mitochondrial metabolism (**A**) and genes of the lipid metabolism (**C**) were analyzed by real-time RT-PCR analysis in HCT116 wild type (WT) cells and HCT116 *IGF2BP2* knockout (KO) cells. Data were normalized to the reference genes 18S, HPRT1 and GAPDH and presented relative to the wild type. Statistical analysis was performed using BioRad CFX Maestro 1.1 Software 2017 (Bio-Rad Laboratories). Data are shown as log2 normalized gene expression; *n* = 3 (triplicates); *p ≤ 0.05, **p ≤ 0.01, ***p ≤ 0.001. **B** Representative Western blot analysis of protein expression in WT and KO cells is shown in duplicates. Tubulin was used as a housekeeping protein. The uncropped Western blot images can be viewed in Fig. S4. **D** WT and KO cells were cotransfected with pLDLR-Luc and pβGal. Luciferase and β-galactosidase activities were measured and calculated as the ratio of luciferase/β-galactosidase enzyme activities and standardized to the WT. Statistical analysis was performed using Student’s *t* test as the data were normally distributed. *n* = 4 (triplicates); *p ≤ 0.05, **p ≤ 0.01, ***p ≤ 0.001. **E** KO cells were transfected with IGF2BP2 (OE) or the control vector. Two days after transfection, overexpression was confirmed by western blot analysis, and gene expression was determined. qPCR data were normalized to the reference genes *18S*, *HPRT1*, *GAPDH* and *ACTB* and presented relative to the cells transfected with the control plasmid. Statistical analysis was performed using BioRad CFX Maestro 1.1 Software 2017 (Bio-Rad Laboratories). Data are shown as log2 normalized gene expression; *n* = 2 (triplicates); *p ≤ 0.05, **p ≤ 0.01, ***p ≤ 0.001. Data shown as box plots (**A**, **C**–**E**). The box is determined by the 25th and 75th percentiles, the whiskers by the 5th and 95th percentiles. Additionally, the median (line), mean (non-filled symbol), and outliers (filled symbols) are shown.

Due to the phenotypic changes in the mitochondrial TCA cycle in the ^13^C-MFA we scanned the significantly differentially expressed genes (DEGs) of the RNA-Seq data (see Table S3) with regard to genes encoding enzymes involved in the TCA cycle, as well as mitochondrial amino acid transporters or transporters of Krebs cycle intermediates. Furthermore, gene expression was confirmed by qPCR. The expression of the following genes was increased in the KO cells compared to the WT: solute carrier family 25 member 10 (*SLC25A10*), uncoupling protein 2 (*UCP2*), isocitrate dehydrogenase (NADP+) 2 (*IDH2*), and pyruvate carboxylase (*PC*) (Fig. 4A). Whereas the expression of mitochondrial pyruvate carrier 1 (*MPC1*) and succinate dehydrogenase complex subunit D (*SDHD*) was decreased (Fig. 4A), no significant change in gene expression was observed for isocitrate dehydrogenase (NADP(+) 3 catalytic subunit alpha (*IDH3A*) (Fig. 4A and Table S3 and S4). On the protein level, SLC25A10 and SDHD expression were not altered. UCP2 expression was distinctly increased and MPC1 expression was slightly decreased in KO compared to WT cells (Figs. 4B and  S4).

Furthermore, the abundance of genes involved in lipid metabolism was measured *via* qPCR. The expression of *SCD*, 3-hydroxy-3-methylglutaryl-CoA reductase (*HMGCR*), and peroxisome proliferator-activated receptor alpha (*PPARA*) was increased, whereas fatty acid synthase (*FASN*) and *ELOVL6* were significantly decreased (Fig. 4C). These findings were consistent with the RNA-Seq data, although *HMGCR* and *ELOVL6* were not significantly altered in the RNA-Seq dataset (see Table S4).

Many lipogenic genes can be induced by a family of transcription factors, i.e., sterol regulatory element-binding proteins (SREBPs). SREBP regulates gene expression as a transcription factor of genes involved in the synthesis of fatty acids, triglycerides and cholesterol [35]. To detect the activity of SREBPs, a luciferase reporter assay was performed using the SRE-containing promoter sequence of the low-density lipoprotein receptor (LDLR) in a luciferase reporter construct. The *IGF2BP2* KO reduced luciferase activity, suggesting lower SREBP activity (Fig. 4D). A rescue experiment using overexpression of *IGF2BP2* in KO cells resulted in a significant reversal in the gene expression of *SLC25A10*, *UCP2*, and *ELOVL6* (Fig. 4E).

### Regulation of MPC1 activity by IGF2BP2 impacts tumor growth

**Fig. 5:**
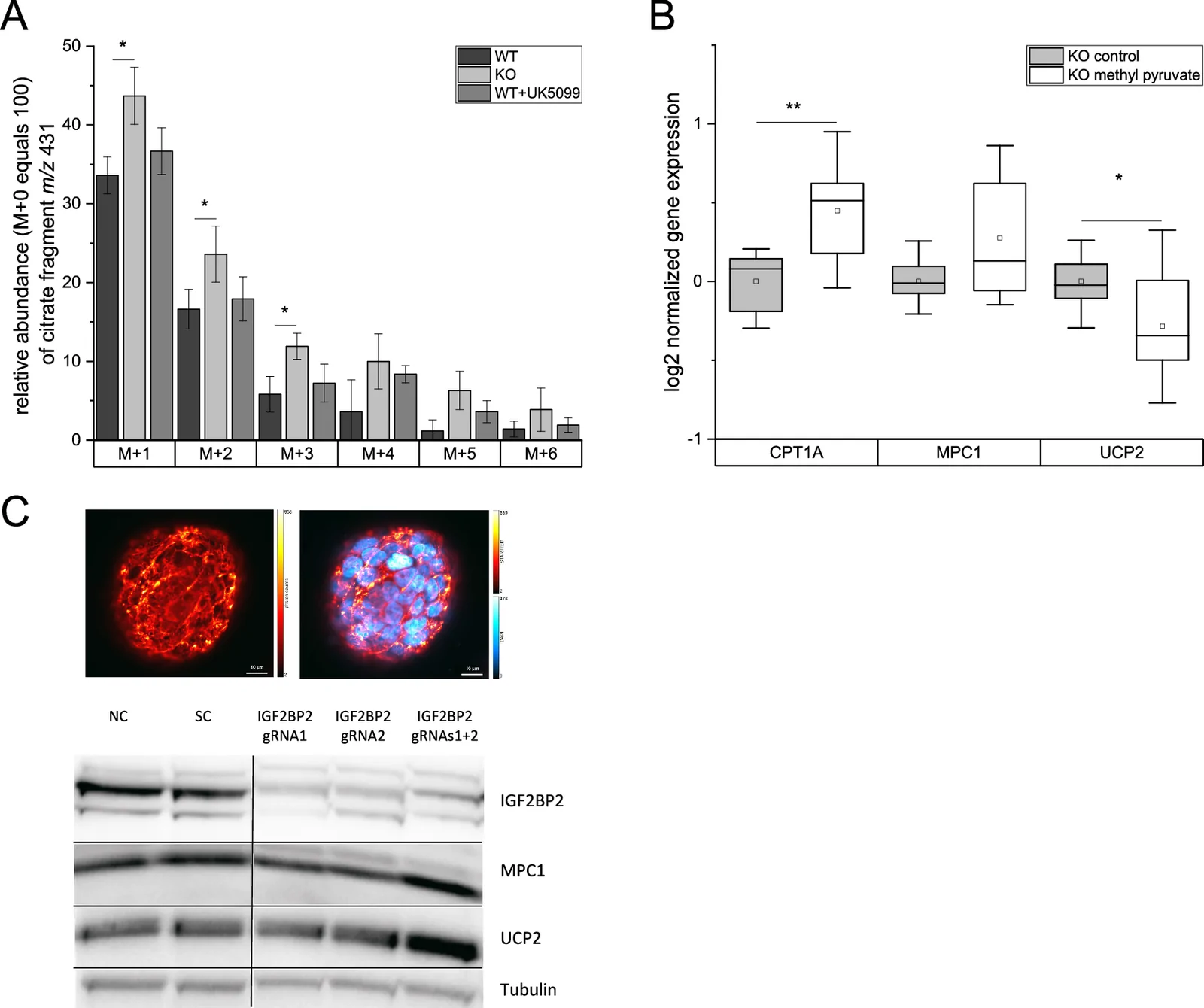
*IGF2BP2* KO reduces MPC1 activity. **A** Mitochondria from WT and KO cells were isolated and fed with U^13^C-glutamine for 2 hours. In addition, the WT cells were also treated with the MPC1 inhibitor UK5099 (200 µM for 1 h; 87 mM stock in DMSO) compared to the control WT cells. Afterward, citrate was extracted and measured by GC-MS. All citrate-specific fragments 431, 459, and 591 were measured *via* GC-MS (*n* = 3). Labeling is shown for fragment 431, representatively. Significance was tested with a Student's *t* test. *p ≤ 0.05, **p ≤ 0.01. **B** Cells in medium containing 50 mM Hepes were treated for 72 h with 5 mM methyl pyruvate (stock 1.11 M in ethanol) or the solvent control. Gene expression was determined by qPCR. Data were normalized to the reference genes 18S and HPRT1 and presented relative to the control. Statistical analysis was performed using BioRad CFX Maestro 1.1 Software 2017 (Bio-Rad Laboratories). Data are shown as log2-normalized gene expression. Data shown as a box plot. The box is determined by the 25th and 75th percentiles, the whiskers by the 5th and 95th percentiles. Additionally, the median (line), mean (non-filled symbol), and outliers (filled symbols) are shown. *n* = 3 (triplicates); *p ≤ 0.05, **p ≤ 0.01. **C** CRC PDO was stained with rhodamine-phalloidin and DAPI to visualize the 3D structure (upper panel). Western blot analysis after CRISPR/Cas9 knockdown of *IGF2BP2* in human CRC PDO, representative images are shown. NC untreated control, SC safe cutter gRNA control, gRNA guide RNA. The uncropped western blot gels can be viewed in Fig. S5.

In order to verify that the lower mitochondrial flux in KO cells is indeed dependent on decreased MPC1 activity, mitochondria of WT and KO cells were isolated and fed with ^13^C-glutamine. Afterward, citrate was extracted and measured by GC-MS. Since cytosolic citrate production *via* IDH3A is not available in isolated mitochondria, mitochondrial citrate production using glutamate competes with mitochondrial citrate production using pyruvate. Indeed, mitochondria from KO cells revealed higher label incorporation compared to WT cells, confirming that glutamate rather than pyruvate was used for citrate production (Fig. 5A). Additionally, treatment of WT cells with the MPC1 inhibitor UK5099 [36, 37] showed a trend toward the citrate labeling pattern observed in *IGF2BP2* KO cells, although the effect remained non-significant and intermediate between WT and KO cells (Fig. 5A).

As methyl pyruvate is able to supply pyruvate into the mitochondria by bypassing the MPC1 [38], we conducted an MPC1 rescue in *IGF2BP2* KO cells. We observed that methyl pyruvate treatment significantly reversed the expression patterns of UCP2 and carnitine palmitoyltransferase 1 isoform A (CPT1A) (Fig. 5B).

Finally, the translational relevance of these findings was confirmed in a colorectal PDO culture. *IGF2BP2* was knocked down in the PDO by CRISPR/Cas9 in bulk cells, resulting in a global knockdown across the PDO culture. The most distinct alterations observed in the HCT116 cells, i.e., in MPC1 and UCP2, were analyzed by Western blot (Fig. S5). Downregulation of IGF2BP2 expression increased UCP2 expression as observed in HCT116 cells (Fig. 5C).

**Fig. 6:**
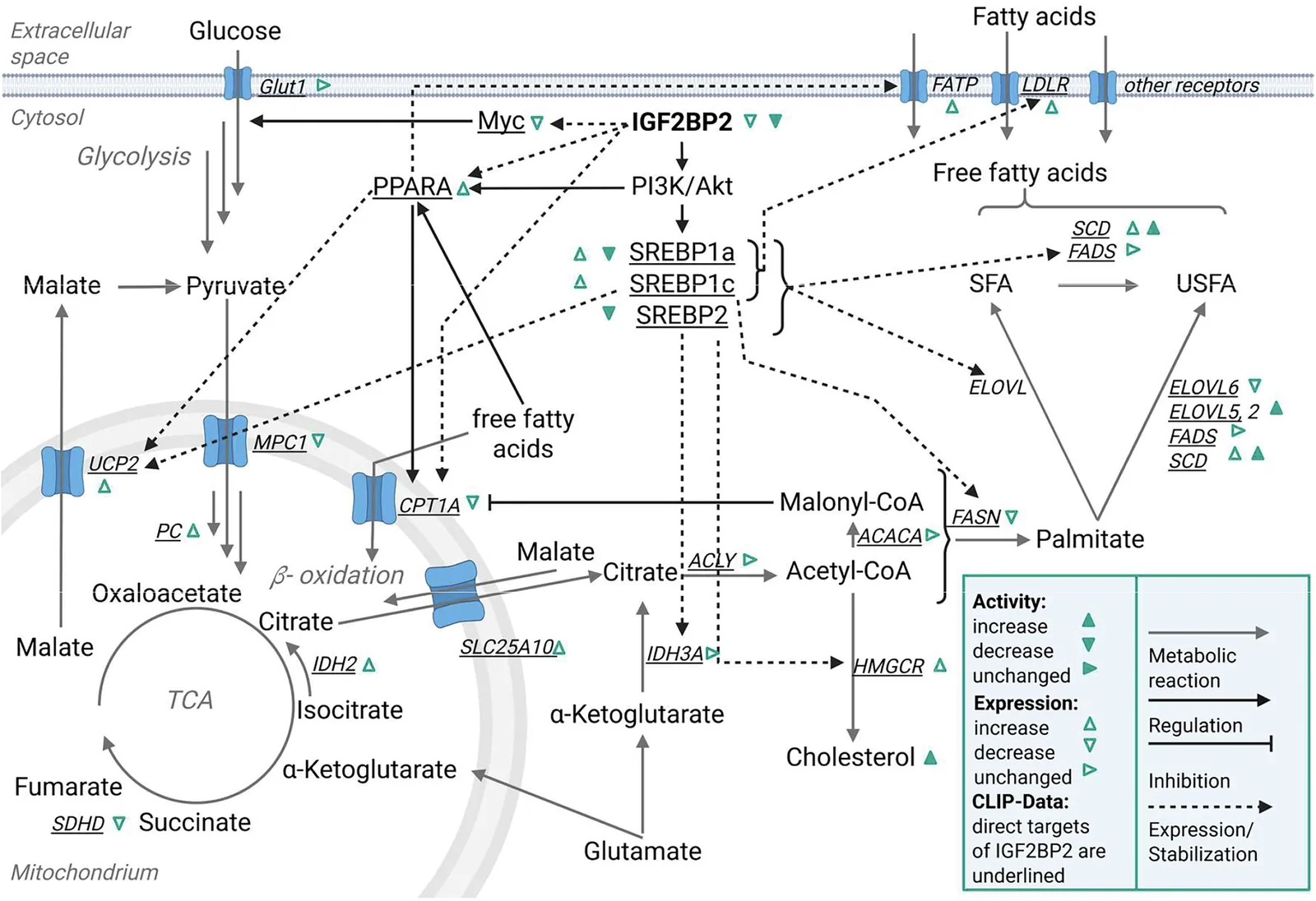
Graphical depiction of the metabolomic and transcriptomic responses to the knockout of *IGF2BP2* in HCT116 colorectal cancer cells found in this study. The arrows depict either a metabolic reaction or transport (gray), a regulatory effect (black), an inhibition (inhibitory arrow) or a transcriptional activation (dotted). The filled-in triangles symbolize an enzymatic activity change, which was observed in ^13^C-metabolic flux analysis, lipidomic analysis, fatty acid distribution or the luciferase assay. The empty triangles represent changes in gene expression observed by RNA sequencing or qPCR. The upright triangle indicates an increase, the downward triangle a decrease, and the sideways triangle an unchanged situation. Potential direct targets of IGF2BP2 according to CLIP data are underlined. Acetyl-CoA carboxylase alpha (ACACA); ATP citrate lyase (ACLY); carnitine palmitoyltransferase 1 isoform A (CPT1A); elongation of very long-chain fatty acids protein (ELOVL); fatty acid desaturase (FADS); fatty acid synthase (FASN); fatty acid transport proteins (FATP); solute carrier family 2 member 1 (GLUT1); 3-hydroxy-3-methylglutaryl-coenzym-A-reductase (HMGCR); insulin-like growth factor 2 mRNA-binding protein 2 (IGF2BP2); isocitrate dehydrogenase (NADP+) 2 (IDH2); isocitrate dehydrogenase (NADP+) 3 catalytic subunit alpha (IDH3A); low-density lipoprotein receptor (LDLR); peroxisome proliferator-activated receptor alpha (PPARA); pyruvate carboxylase (PC); mitochondrial pyruvate carrier 1 (MPC1); MYC proto-oncogene (Myc); saturated fatty acids (SFA); solute carrier family 25 member 10 (SLC25A10); stearoyl-CoA desaturase (SCD); sterol regulatory element-binding proteins (SREBP); succinate dehydrogenase complex subunit D (SDHD); tricarboxylic acid cycle (TCA); uncoupling protein 2 (UCP2); unsaturated fatty acids (USFA). Figure was generated in BioRender.com.

In summary, the diverse effects of IGF2BP2 now come into play (as depicted in Fig. 6), raising the question of which of the above factors are potential direct targets of IGF2BP2. To this end, we used publicly available CLIP data of IGF2BP2 and screened all the factors listed in Fig. 6. Based on the CLIP data, the vast majority of these factors are potential direct RNA targets of IGF2BP2 (see Table S4 and Fig. 6): CPT1A; FASN; IDH2; IDH3A; LDLR; MPC1; MYC; PC; PPARA; SCD; SDHD; SLC25A10; SLC27A2/FATP2; SREBF1; UCP2.

## Discussion

IGF2BP2 has been extensively studied in different cancer types [6, 7, 11, 24, 39–41]. Its increased expression is associated with poor patient prognosis in CRC and liver cancer [2, 5]. IGF2BP2 is known to affect localization, stability, modification, and processing of a variety of mRNAs, thereby influencing cellular metabolism. Previous studies focused only on individual metabolic pathways in various types of cancer. They examined three critical pathways: aerobic glycolysis, lipid and amino acid metabolism [1, 24, 42, 43]. The current study visualizes how IGF2BP2 affects the overall metabolism of colorectal cancer cells. For this purpose, steady-state ^13^C-MFA of HCT116 WT and *IGF2BP2* KO cells [7] as a model was used as a starting point.

The *IGF2BP2* KO cells were shown to have generally reduced metabolic fluxes into and within the mitochondria. Thus, mitochondrial energy production is likely reduced due to lower fluxes. A reduced OXPHOS after *IGF2BP2* KO was reported to be caused by a reduced activity in complex 1 and 4 in glioblastoma stem cells [24]. In the current study, the total amount of adenine nucleotides as well as the energy charge of the adenylate system [44] were unchanged between KO and WT cells, which is in concordance with our previous study also showing an unaltered total ATP production, but a shift from mitochondrial ATP production to aerobic glycolysis in the same KO cell line [5]. Further, Janiszewska et al. reported an unchanged total ATP amount after knockdown of *IGF2BP2* [24]. However, in some tissues, as shown in brown adipocytes, a KO of *IGF2BP2* does not affect mitochondrial oxidative capacity [17], suggesting tissue or cell-type-specific effects. Taken together, IGF2BP2 induces energy production *via* OXPHOS, and in the absence of IGF2BP2 expression, there is a rerouting to aerobic glycolysis. This might be mechanistically caused by translocation of essential mitochondrial RNA templates or trafficking of transcripts for the energy production by IGF2BP2 [4].

In addition to the energy shift, the MID data comparing KO and WT suggested reduced transport reactions between mitochondria and the cytosol in KO. The MIDs of citrate and malate with the different tracers indicate that the TCA metabolites in the KO cells are predominantly produced *via* the glutamine carbon and not *via* the glycolysis carbon. Further, the altered glutamine metabolism confirms a rerouting of glutamine almost exclusively into the mitochondria and a reduction of glycolytic carbon intake into the mitochondria. In acute myeloid leukemia cells, reduced glutamine uptake was observed after *IGF2BP2* knockdown, resulting in decreased levels of TCA cycle intermediates [45]. Weng et al. [45] also showed that IGF2BP2 has a stabilizing and translation-promoting effect on m6A reader-modified target transcripts involved in glutamine metabolism in acute myeloid leukemia cells, particularly MYC proto-oncogene (MYC), solute carrier 1 family 1 member 5 (SLC1A5) and glutamate pyruvate transaminase 2 (GPT2) [45]. The oncogene MYC is a direct target gene of IGF2BP2 [46] and regulates both glutamine metabolism, *via* SLC1A5 and glutaminase (GLS1), as well as glucose metabolism [45]. While consistent with these findings, our ^13^C-MFA revealed a paradoxical pattern: despite a downward trend in glutamine uptake, TCA cycle intermediates exhibited increased labeling from U¹³C-glutamine, indicative of enhanced oxidative glutamine catabolism to CO_2_. This metabolic phenotype, characterized by diminished substrate import coupled with heightened intracellular oxidation, represents a novel finding that challenges previous hypotheses proposed by Weng et al. [45]. The complete oxidation of glutamine provides the highest ATP yield out of all possible glutamine fates. Glutamine could also be metabolized *via* reductive carboxylation or normal anaplerosis. If the latter were the case, enhanced labeling of palmitate by labeled U^13^C-glutamine would be observed. Another possibility would be glutaminolysis, where a label increase in lactate would occur [47]. Neither of these was observed. The complete oxidation might explain the energetic maintenance of the KO cells despite the overall reduction of the metabolic fluxes [47]. This points to shifts in transporter reaction activities and was reinforced by the citrate labeling pattern from isolated mitochondria. There, a complete oxidation of glutamine could be seen by a much higher ^13^C-glutamine incorporation into citrate, especially in the M + 3 isotopomer [37, 48].

IGF2BP2 has been reported to target transcripts of electron-transfer chain components and other mitochondrial components [5, 17, 49]. The CLIP data suggest binding of IGF2BP2 to mRNAs associated with mitochondrial respiration. Previously, gene expression data of the same KO cells showed that the OXPHOS genes *NDUFAF4*, *CYB5B* and *COX16* were reduced [5]. Concordantly, hematopoietic stem cells from young *Igf2bp2* KO mice exhibit age-related changes, partly due to a reduction in mitochondrial gene expression, leading to a subsequent loss of mitochondrial function [50]. In addition, the current study demonstrates that IGF2BP2 further affects mitochondrial genes encoding enzymes and transporters, such as *UCP2*, *SLC25A10*, *PC*, *SDHD*, *MPC1*, and *IDH2*, thereby underscoring changes in the metabolic phenotype. These transporter regulations seem to be, at least in parts, responsible for the IGF2BP2-induced changes in the mitochondrial fluxes. Indeed, maintenance of the TCA cycle *via* glutamine can be supported by increased UCP2 [51]. PPARs [52] and SREBPs [53] can regulate UCP gene transcription. Additionally, we observed increased expression levels of *SLC25A10*, which encodes a mitochondrial antiporter that exchanges cytosolic malate for mitochondrial citrate [54]. Both UCP2 and SLC25A10 transport malate in opposite directions. Combined with the preferential complete oxidation of glutamine, this seems to indicate that outward transport of malate and inward transport function as a cycle to allow the cell to continue to export citrate for fatty acid synthesis. SLC25A10 is reported to influence de novo fatty acid synthesis *by mediating* malate transport to supply cytosolic citrate [54]. PC provides oxaloacetate from pyruvate for the TCA cycle as an anaplerotic reaction. Again, the increased expression of *PC* supports the hypothesis of complete glutamine oxidation. It has been suggested that PC expression is also promoted by PPARɑ [55]. Thus, the increased expression of *PPARɑ* underlines our findings. The decreased SDHD gene expression confirms both the overall lower mitochondrial flux depicted in the KO flux map and the increased PC activity [56].

Another key finding that supports glutamine rerouting is the modest reduction in *MPC1* expression. While both IGF2BP2 and MPC1 are involved in metabolic regulation, to date, no regulatory or functional link between them has been reported. According to CLIP data, *MPC1* is a direct target of IGF2BP2. MPC1 is responsible for the transport of glucose-derived pyruvate into the mitochondria [57]. A shift in glutamine utilization toward complete oxidation was also observed in a ^13^C-MFA of C2C12 myoblasts with *MPC1/2* knockdown [58] and in a labeling study that inhibited MPC1 with UK5099 in SFxL cells [37]. These metabolic shifts could account for the overall decreased metabolic fluxes in the mitochondria in the *IGF2BP2* KO cells, as seen in the ^13^C-MFA and labeling pattern of citrate in isolated mitochondria. The increased label incorporation from citrate derived from U^13^C-glutamine in the KO cells mirrors the effect of the MPC1 inhibitor UK5099 as described for SFxL cells [37]. However, only minor changes in *MPC1* levels could be observed after *IGF2BP2* knockdown, suggesting that changes occur primarily in transporter activity rather than expression due to IGF2BP2 modulation. Methyl pyruvate treatment in KO cells reversed the expression of *UCP2* and *CPT1A*, implicating MPC1-dependent metabolic rewiring. While CLIP data suggest direct IGF2BP2 binding to *UCP2* mRNA, evidence for a functional *IGF2BP2-UCP2* axis is currently lacking. IGF2BP2 is reported to decrease mRNA abundance, shorten the half-life and reduce mRNA polysomal abundance of *CPT1A* in *IGF2BP2* CRISPR AML12 cells [16]. Notably, no existing study has examined the potential interconnections among UCP2, CPT1A, and MPC1 under IGF2BP2 regulation.

The malignant changes in cancer cell metabolism are often accompanied by an altered lipid metabolism in different cancer types, such as colorectal carcinoma (CRC) [32, 59], colon cancer [60], esophageal adenocarcinoma [61], liver cancer [62], non-small cell lung cancer [63] and breast cancer [64]. IGF2BP2 is known to alter lipid homeostasis through post-transcriptional mechanisms [3, 65, 66]. The altered acetyl-CoA production in the KO flux map compared to WT, and the shift in MID of palmitate, led us to conclude that IGF2BP2 alters fatty acid metabolism. The lipidome data confirmed clear differences between KO vs. WT, which might affect survival, proliferation, or cell-cell communication [59]. The impact of these changes varies across species and culture conditions in the literature [59].

Regarding lipogenic gene expression in the *IGF2BP2* KO cells, the expression of *FASN* was decreased, while *ACLY* and *ACACA* showed no significant difference, although all three have been described to have increased activity in cancer cells [67]. The de novo lipogenesis is associated with enhanced saturation and increased *FASN* in HCT116 cells [68]. An overexpression of *FASN* is negatively associated with CRC patient survival [69], and a knockdown of *FASN* resulted in a reduced cancer invasion and spread in cell lines [70].

FASN, ACACA and ACLY are controlled by SREBP [67, 71–73]. SREBPs have been described to promote both colon cell proliferation and the ability to form tumor spheroids [35]. The increased expression but lower activity of SREBP might be explained by cholesterol-mediated negative feedback. In HepG2 cells, the overexpression of p62, a splice variant of IGF2BP2, induced an increase in SREBP1 activity, although no correlation between *p62* and *FASN* was observed in human liver cancer samples [19]. SREBP is also reported to increase the LDLR in the cell membrane [74, 75]. The increased expression of *LDLR* in the KO cells may lead to a higher uptake of fatty acids as building blocks, indicating a shift to diet-derived fatty acids in the KO cells compared to the WT.

SREBPs are further involved in the promotion of ELOVL6 [76] and SCDs [77]. ELOVLs and SCDs produce a vast spectrum of fatty acids starting from palmitate [67]. The elevated fatty acid ratios of monounsaturated and unsaturated palmitate and stearate of the KO cells provide evidence for an estimated increase in SCD activity [32], which was further confirmed by higher gene expression. Complete inhibition of SCD activity, however, is reported to block cancer cell proliferation and reduce survival [78] and silencing *SCD* with siRNA in HCT116 cells resulted in induced apoptosis [79] and reduced viability in HCT116 xenografts [80]. In hepatocyte-specific *Igf2bp2-*deficient mice, however, no difference in *SCD*, SREBP1c and SREBP2 was reported [16], although hepatocytic overexpression of IGF2BP2 induced SREBP activity [19]. ELOVL6 is known to be induced by IGF2BP2 in hepatocytes [20]. Concordantly, gene expression decreased, although estimated enzyme activity was higher in KO colon cells. A study investigating CRC tissues reported increased ELOVL6 activity, expression and fatty acid elongation in tumor tissue [81]. Still, specific ELOVL involvement seems to be cancer-type-specific [59].

IGF2BP2 also stabilizes *CPT1A* and PPARɑ polypeptides [16]. PPARɑ is involved in the regulation of lipid oxidation, activation, and oxidation of fatty acids, lipolysis, ketogenesis, and storage of triglycerides [82–84]. PPARɑ decreased following a hepatocyte-specific Igf2bp2 deletion in mice [16]. In our study, PPARα was significantly increased in KO cells, likely due to reduced activity of the downstream PI3K/AKT axis, which in turn regulates PPARα expression [18, 85]. PPARɑ is also responsible for the fatty acid uptake into the cells through a transcriptional promotion of fatty acid transport proteins (FATP) [86], some of which were increased in the KO cells. CPT1A, the transporter that catalyzes the rate-limiting step of *β*-oxidation, translocates fatty acids into the inner mitochondrial membrane [82]. The decrease of *CPT1A*, together with the unchanged glucose-derived palmitate label, suggests a reduced *β*-oxidation and an increased dietary uptake of fatty acids. Taken together, a higher fatty acid uptake but a lower *β*-oxidation of lipids occurs in the KO cells. Most of these genes controlling lipid moieties can be linked to IGF2BP2 as potential direct RNA targets based on CLIP data.

Overall, this suggests that the increase in the amount of specific lipid species in the *IGF2BP2* KO cells is more likely due to increased uptake and elongation than to increased fatty acid production. Furthermore, increased saturation is correlated with increased lipogenesis in HCT116 cells [68], which is in turn correlated with cancer cell metabolism. Thus, IGF2BP2-expressing cells show a more cancer-like lipogenic phenotype in comparison to cells lacking IGF2BP2.

In summary, the current study comprehensively depicts the complex influence of IGF2BP2 on primary and secondary metabolism. IGF2BP2 has been shown to have a major impact on tumor cell metabolism, including mitochondrial metabolism and cancer-specific changes in lipid metabolism, both of which promote tumor cell survival.

However, this study has potential limitations. Since the experiments were conducted in vitro, further studies using animal models or clinical samples are warranted. Additionally, we used one single cell line, HCT116, which carries, among other alterations, a KRAS G13D mutation. Mutations are a characteristic feature of CRC, altering the cancer phenotype [87], and need to be taken into consideration. Even though 50% of patients with metastatic CRC carry a RAS mutation [88], the biomarkers known so far do not capture the diversity of inter- and intra-tumor heterogeneity [87]. Moreover, IGF2BP2 expression in CRC appears to be independent of BRAF and KRAS mutation status, as previously reported [5]. Still, this study provides a comprehensive overview of metabolic changes by IGF2BP2 in the metabolism of colon cancer cells. It remains to be determined which specific mechanisms IGF2BP2 uses to mediate these metabolic changes by interacting with different targets, and to verify causal relationships.

## Material and methods

### Cell lines

The HCT116 WT cell line was originally derived from the colon cancer tissue of an adult male. Cell line authentication was conducted by STR/DNA profiling. For cell line authentication, the Cell Line Authentication Service by Eurofins Genomics was used. DNA was isolated from a cell pellet. Genetic characteristics were determined by PCR-single-locus-technology. 16 independent STR loci, D8S1179, D21S11, D7S820, CSF1PO, D3S1358, TH01, D13S317, D16S539, D2S1338, AMEL, D5S818, FGA, D19S433, vWA, TPOX, and D18S51 were investigated using the AmpFlSTR® Identifiler® Plus PCR Amplification Kit (Thermo Fisher). In parallel, positive and negative controls were included, yielding correct results. Results were compared to a reference sample and known profiles. Mycoplasma testing was performed regularly *via* PCR. For that purpose, cells were grown to confluence over several days in antibiotic-free medium. A PCR was performed with the supernatant of the cells (Primer forward 5′-3′: GGCGAATGGGTGAGTAACACG, Primer reverse 5′-3′: CGGATAACGCTTGCGACCTATG). Positive samples showed a distinct 500 bp band on the agarose gel. The WT and HCT116 *IGF2BP2* KO cells were cultured in Dulbecco´s modified Eagle´s medium (DMEM, #21969-035, Gibco) supplemented with 10% heat-inactivated fetal calf serum (FCSi, #P30-3306, PAN-Biotech), 2 mM L-glutamine (#X0551-100, Biowest), 100 U/mL penicillin, and 100 μg/mL streptomycin (Pen/Strep, #15070-063, Gibco). The IGF2BP2 KO cell clone was generated in a previous study by CRISPR/Cas9-mediated KO [2]. Clone KO#1 from the latter study was used for all experiments in the current study. Cells were grown in 95% humidity, at 5% CO_2_ and 37 °C. Cell line authentication, as well as scheduled testing for the absence of mycoplasmas, was conducted as previously described [5]. The confirmation of the *IGF2BP2* KO was achieved by Western blot analysis (Fig. S6). Overexpression of *IGF2BP2* was performed as described previously [2]. In brief, 0.125 Mio cells were seeded into a 24-well plate. Transfection was conducted 24 h later with 0.75 µl Lipofectamine 3000 (#L3000015, Thermo Fisher) and 500 ng plasmid DNA per well according to the manufacturer’s instructions. After 48 h, overexpression was confirmed by Western blot and gene expression analysis was conducted.

### Patient-derived organoid culture

In this study, one patient-derived organoid culture from a CRC patient was used. Informed consent was obtained from all human subjects included in the study. The study was approved by the local ethics committee of the University of Halle (#2019-037). Upon receipt of the resected sample, fatty tissue was discarded, and the tumor sample was cut into small pieces with a scalpel and tweezers. For digestions, an enzyme mix according to the tumor dissociation kit protocol (Miltenyi Biotec #130-095-929) was added, and dissociation was performed using the gentleMACS Dissociator (#130-093-235), followed by an incubation at 37 °C for 30 minutes with the Macsmix Tube Rotator (#130-090-753). Afterward, gentleMACS Dissociator was used again, and the sample was incubated at 37 °C for 30min. SmartStrainer (#130-098-462) was attached to a new 50 ml tube, and the cell suspension was applied. The cell suspension was centrifuged for 10 min at 650 RCF, and the supernatant was discarded.

For red blood cell lysis, RBC lysis solution (#130-094-183) (10×) was diluted 1:10 with ddH_2_O and added to the cell pellet, vortexed for 5 s and incubated for 2 min at room temperature, centrifuged (10 min, 650 RCF), and the supernatant was discarded. The pellet was resuspended in an appropriate volume of BME, and the organoid BME mixture was seeded into preheated 12-well plates as 10–20 µl droplets. The plate was gently flipped and incubated for 20–30min at 37 °C for polymerization. Solidified droplets were carefully overlaid with 37 °C preheated organoid medium, including 1.25 µg/ml Amphotericin. Amphotericin was discarded after the first week of culture.

For organoid dissociation, the organoid pellet was resuspended in 1 ml TrypLE (#12604013, Gibco), dissociation was stopped by adding 10 ml DMEM at room temperature and centrifuged at 500 × *g* for 10 min. Up to 1 ml of supernatant was discarded, and the pellet was resuspended, strained with a FACS tube strainer (#Corning Falcon™ 352235) and counted.

For bulk CRISPR-Cas9 genome editing, a ribonucleoprotein (RNP) electroporation approach was used. The following guide RNAs (gRNAs) were used: safer cutter gRNA 5’-GAATAATCATAACAAGGATA-3’ as a control guide and Hs.Cas9.IGF2BP2.1.AA 5’-ATATGTGACGTTGACAACGG-3’ and Hs.Cas9.IGF2BP2.1.AC 5’-GTGCGCTGAGGTTCAACCCT-3’ for IGF2BP2 KO as a single guide and also in combination (all from IDT Biologica).

Alt-R CRISPR-Cas9 sgRNA was prepared according to the manufacturer’s protocol to a final concentration of 44 µM. To form the RNP complex, 0.3 µl Alt-R Cas9 enzyme was mixed with 0.2 µl Resuspension Buffer R (Neon Kit system) to a total volume of 0.5 µl. Afterward, 0.5 µl sgRNA was added to the diluted Alt-R Cas9 enzyme, and the mixture was incubated at room temperature for 10-20minutes. Cells were resuspended by adding 9 µl of Resuspension Buffer R per electroporation, mixed with 1 µl RNP complex, and electroporated using the Neon NXT system set to 1600 V, 10 ms, and 3 pulses.

### Phalloidin staining

For immunofluorescence staining of organoids, the medium was removed from the organoid culture and gently washed two times with 1× PBS. 30–40 organoid Matrigel domes were fixed in a 50 ml tube with 15–20 ml of 4% PFA for 30 min at room temperature on an orbital shaker.

Afterward, organoids were allowed to settle at the bottom of the tube by gravity ( ~ 10–15 minutes). 15–20 ml of 1× PBS was added and was let to rest for 20 mins at room temperature. The procedure was repeated three more times. Blocking was performed with 0.5 ml blocking buffer (5% BSA + 0.5% Triton X-100 in 1× PBS) overnight at 4 °C. Direct conjugated antibodies diluted in blocking buffer were incubated at 4 °C overnight. Then, samples were washed three times with 1× PBS for 15 minutes each wash. DAPI was used for nuclear staining counterstaining and incubated at room temperature for 15-20 minutes. Samples were washed three times with 1× PBS for 10-15 minutes each wash. Afterward, samples were imaged on a STED- and confocal microscope (Stedycon, Abberior Instruments) based on an epifluorescence microscope (Olympus IX83).

### Western blot analysis

To confirm the *IGF2BP2* KO, HCT116 cells were lysed on ice with ice-cold RIPA buffer (Tris-HCl 50 mM, NaCl 150 mM, Triton X-100 1%, sodium deoxycholate 1%, SDS 0.1%) with protease inhibitor (cOmplete® mini protease inhibitor cocktail, #04693124001, Roche Diagnostics). After lysis, the cell debris was removed *via* centrifugation at 14,000 × *g* for 10 min at 4 °C. Determination of the protein amount was conducted with the Pierce™ BCA Protein Assay-Kit (#23227, Thermo Scientific) according to the manufacturer´s protocols and the use of the FLUOstar OPTIMA plate reader (#413-0505, BMG Labtech). After determination of the concentration, the samples were mixed with 10x Bolt™ Sample reducing agent (#B0004, Invitrogen) and 4× Bolt™ LDS sample buffer (#B0007, Invitrogen) and heated the samples for 5 min at 95 °C. The PageRuler Plus Prestained Protein Ladder (#26619, Thermo Scientific) was used to determine protein sizes. SDS-polyacrylamide gel electrophoresis (SDS-PAGE) was realized using the PowerEase™ Touch 350 W Power Supply (200 V, 3000 mA, 350 W, 20 min; Thermo Fisher Scientific, #PS0351), the Mini Gel Tank (Thermo Fisher Scientific, #A25977), Bolt™ MOPS SDS Running Buffer (Thermo Fisher Scientific, #B0001) and Bolt™ 4–12% and Bis-Tris, 1 mm Mini-Protein-Gels (Thermo Fisher Scientific, #NW04120BOX). The iBlot3 Western Blot Transfer System (25 V, 6 min; Thermo Fisher Scientific, #IB31001) and iBlot3™ Transfer Stacks (midi, NC, Thermo Fisher Scientific, #IB33001) were used to transfer separated proteins. The membrane was blocked for 2 h using Rockland Blocking Buffer (#MB-070, Rockland Immunochemicals). Antibodies used were specific for IGF2BP2 [89] and α-tubulin (Sc-32293, Santa Cruz). Both primary antibodies were diluted 1:1000 in Rockland Blocking Buffer, and the membrane was separately incubated overnight at 4 °C. Secondary Peroxidase-conjugated antibodies were diluted 1:5000 (#111-035-144, Jackson Immuno Research Laboratories Inc) and 1:2500 (#715-035-150, Jackson Immuno Research Laboratories Inc) in 5% milkpowder in TBS-T. The membrane was incubated for 1 h at room temperature. For chemiluminescence detection, the SuperSignal™ West Pico Plus Chemiluminescent Substrate (#34580, Thermo Scientific) was used according to the manufacturer’s guidelines. Detection was performed with the Octoplus QPLEX Analyzer (nhDiagnostics, Halle, Germany). Additional antibodies used: MPC1 (Cell Signaling; 14462S), SDHD (abcam; ab189945), SLC25A10 (proteintech; 12086-1-AP), UCP2 (Cell Signaling, 89326S).

### Isotopic tracer experiments and extraction of intracellular metabolites

For the tracer experiment, DMEM (#P04-01549, PAN-Biotech) was supplemented with 1% of FCSi, 1% Pen/Strep, 2% Insulin-Transferrin-Selenium (ITS, #41400-045, gibco), unlabeled glucose (5 g/l, #G-75287, Sigma-Aldrich), and 2 mM L-glutamine (#X0551-100, biowest) or the labeled tracers 1,2^13^C-glucose (99%, #453188, Sigma-Aldrich Chemie GmbH), U^13^C-glucose (99%, #389374, Sigma-Aldrich Chemie GmbH) or U^13^C-glutamine (99%, #CLM-1822-H, Cambridge Isotope Laboratories), respectively. Cells were maintained in the unlabeled medium for two passages before starting the experiment. 50,000 cells were seeded in six-well plates. After 24 h, incubation with the medium was changed to medium containing either the respective labeled tracers or the unlabeled control. After 72 h of cultivation, the medium was exchanged daily until day 8. Supernatants of glucose and glutamine control wells were collected before and after medium exchange along the growth phase for the determination of exchange rates. On day 8, the supernatant was removed, 600 µl of −20 °C cold methanol per well was added and incubated on ice for 15 min. The cells were collected by scraping. 600 µl of water was added, and the cell suspension was transferred to a new 2 ml reaction tube. In total, 600 µl of chloroform was added, and the samples were vortexed for 15 min. The phases and cell debris were separated after centrifugation for 15 min at 4 °C by 14.000 × *g* and stored at −80 °C till further use.

### Determination of exchange rates

For the determination of the glucose consumption, the supernatant, which was collected as stated above, was diluted 1:4 in water. The 200 ml aqueous buffer containing imidazole and MgCl_2_, pH was adjusted to 6.9. The measurement buffer additionally contained 75 mg NAD^+^ (#10127973001, Roche) and 30 mg ATP (#10127523001, Roche) in 50 ml buffer. 10µl of the diluted sample was transferred to a 96-well plate, and 10 µl of glucose-6-phosphate dehydrogenase (#10165875001, Roche), diluted 1:20 in buffer, was added, as well as 270 µl measuring buffer. The background was measured with a BioTek microplate reader ELx 808 (BioTek Germany; Bad Friedrichshall) at 590 nm for 15 min. Then, 10 µl 1:20 hexokinase (#11426362001, Roche) diluted in buffer was added per well. The absorbance was measured at 590 nm for 45 min. The glucose concentration was calculated according to the Beer-Lambert law.

To determine the exchange rates of amino acids and lactate, the concentrations in the fresh and used supernatants were measured at different time points using a GC/Q-TOF Agilent 7890B/7200 (Agilent, Waldbronn, Germany). The supernatant was diluted 1:100 in water, 100 µl were evaporated under vacuum. The samples were dissolved in 30 µl acetonitrile (ACN), derivatized with 40 µl *N*-*tert*-Butyldimethylsilyl-*N*-methyltrifluoroacetamide (MBDSTFA, #77626, Sigma-Aldrich) at 60 °C for 1 h and then directly measured three times. The derivatization was done and scheduled by an Autosampler (Gerstel GmbH & Co. KG, Germany). A HP-5MS column (Ultra Inert 30 m, 0.25 mm, 0.25 µm, Agilent, Santa Clara, USA) was equipped in the GC system, and the helium flow was set to 1 ml/min. The temperature gradient was set to 60 °C for 12 min, followed by a linear ramp of 20 °C/min to 280 °C and held at this temperature for 35 min. The MS was outfitted with an electron impact (EI) source operating at 70 eV and 230 °C source temperature. The liner and transfer line were held at 250 °C. The raw data were processed by MassHunter Quantitative Analysis software (Agilent, B.10.00, Santa Clara, USA).

### Determination of mass isotopomer distribution

The mass isotopomer distribution was performed on a CG 7890B coupled with a 5977B MSD with a 7693 autosampler (Agilent, Waldbronn, Germany), equipped with a HP-5MS column (Ultra Inert 30 m, 0.25 mm, 0.25 µm, Agilent, Santa Clara, USA). The helium flow was 1 ml/min. The liner, transfer line, and source temperature were set to 250 °C, 250 °C and 230 °C, respectively. The MS was outfitted with an EI source operating at 70 eV.

For the MID of the amino acids, the cell debris was hydrolyzed with 800 µl of 6 N HCl and shaken at 99 °C overnight. 5 µl of the hydrolysate were evaporated under N_2_, dissolved in 30 µl ACN and derivatized with 40 µl MBDSTFA at 60 °C for 1 h. The temperature gradient was set to 60 °C for 2 min, followed by a linear ramp of 20 °C/min to 280 °C and held at this temperature for 5 min.

For the MID of the organic acids 300 µl of the polar phase was dried, incubated with 30 µl 1 mg/ml methoxyamine (MeOX; #226904, Sigma-Aldrich) in pyridine at 60 °C for 1 h and derivatized with 40 µl MBDSTFA for 1 h at 60 °C. The temperature gradient was set to 60 °C for 2 min, followed by a linear ramp of 10 °C/min to 300 °C, and held at the same temperature for 2 min.

For the MID of sugars, the cell debris was hydrolyzed with 50 µl of 6 N HCl for 30 min at 30 °C. 250 µl water was added, and samples were incubated for 1 h at 100 °C. 80 µl were evaporated under N_2_ and incubated with 30 µl MeOX in pyridine at 60 °C for 1 h and derivatized with 40 µl *N*,*O*-Bis(trimethylsilyl)trifluoroacetamide (BSTFA, #25561-30-2, Macherey-Nagel) for 1 h shaking at 60 °C. The temperature gradient was set to 60 °C for 2 min, followed by a linear ramp of 10 °C/min to 325 °C and held at this temperature for 10 min.

For the MID of palmitate 100 µl of the chloroform phase was evaporated under N_2_, and 40 µl of trimethylsulfonium hydroxide (#701520.201, Macherey-Nagel) was added. 1 µl of this solution was directly measured. The temperature gradient was set to 60 °C for 2 min, followed by a linear ramp of 10 °C/min to 300 °C and held at this temperature for 2 min.

The raw data were processed by MassHunter Qualitative Analysis software (Agilent, B.10.00, Santa Clara, USA).

Mass isotopomer data were corrected for natural abundance and original unlabeled biomass using the Perl script iMS2flux [90].

### Determination of glutathione and AMP, ADP, ATP

100 µl of the polar phase of the unlabeled controls was used to determine ratios of GSH, GSSG, AMP, ADP and ATP *via* LCMS/MS Shimadzu Nexera X2/AB Sciex QTRAP 5500 (Shimadzu (Duisburg)/AB SCIEX (Darmstadt)).

Separation was performed at 40 °C using ion pairing chromatography on a 2.7 μm RP UPLC column (EC 150/2 Nucleoshell RP18, 150 mm × 2 mm, Macherey-Nagel, Düren, Germany). The mobile phase consisted of eluent A containing 10 mM tributylamine (#102-82-9, Sigma-Aldrich) acidified with glacial acid (#5.33001.0052, Sigma-Aldrich) to pH 6.2 and eluent B containing acetonitrile. It was pumped at a flow rate of 0.4 ml/min using different isocratic and gradient elution steps. From the injection, the column was eluted 2 min with 2% eluent B, followed by a gradient of eluent B within 16 min from 2% to 36%, a consecutive gradient of 3 min from 36% to 95% and a final isocratic elution with 95% for 4.5 min. To restore the elution of 2% B, a gradient elution from 95% to 2% B over 0.01 min was used. A last isocratic elution of 2% B ran for 5 min (total run time 31.01 min). MS/MS data were acquired in scheduled multiple reaction monitoring mode using the software Analyst version 1.6.3 (AB Sciex GmbH, Darmstadt, Germany).

### Determination of fatty acid distribution

Three Mio WT and KO cells, maintained in DMEM with 10% FCSi, were harvested in quadruplicates, centrifuged, and the supernatant was removed. The cell pellets were lyophilized over 48 h (Epsilon 2–4 LSCplus freeze dryer, Christ) and the dry weight was determined. The dried cell pellets were extracted as described above, adding 30 µl methylnonadecanoate as internal standard. 10 µl of the chloroform phase was transferred into a conical glass vial, dried under nitrogen, and 80 µl trimethylsulfonium hydroxide was added, and the vial was closed. Fatty acid methyl esters were measured using the same machine and settings as those described for the MID. The temperature gradient was set to 60 °C for 2 min, followed by a linear ramp of 10 °C/min to 300 °C and held at this temperature for 2 min.

The raw data were processed by MassHunter Quantitative Analysis software (Agilent, B.10.00, Santa Clara, USA), and normalized to the internal standard and the dry weight of the cell pellet.

### Metabolic network model

For the ^13^C-MFA a compartmentalized metabolic network model of HCT116 cells was constructed by reviewing the respective literature and online databases [91–98]. The complete model can be consulted in Table S1. The model consists of 39 biochemical reactions and 19 transport reactions in the three compartments: cytosol, mitochondria, and extracellular medium. It includes reactions for the glycolysis, fermentation, OXPHOS, pentose phosphate pathway, TCA cycle, anabolic and catabolic reactions, amino acid metabolism, and a lumped biomass reaction. The biomass reaction was calculated using the published biomass equation by Niklas et al. [99]. Mixing reactions were implemented to model compartment-specific pools [100]. The catabolic reactions were calculated as the uptake of malate and acetyl-CoA instead of the uptake of single, unlabeled amino acids [101]. Labeled amino acids were integrated directly into the biomass. Transport between the compartments was modeled as simple passive transport reactions. To include the influence of the unlabeled carbon of atmospheric CO_2_, a CO_2_ exchange reaction was added [102].

### Metabolic flux analysis

^13^C-metabolic flux analysis was performed using the MFA Suite™ Isotopomer Network Compartmental Analysis (INCA) 2.1 [26], a Matlab-based flux estimation tool using the elementary metabolite units (EMU) framework [103, 104].

Fluxes were estimated by minimizing the variance-weighted sum of squared residuals (SSR) between the experimental data (exchange rates, mass isotopomer distribution) and the model's predicted data using non-linear least-squares regression [105]. The calculation was performed using a parallel approach, with three experimental labeling data sets. The flux values were calculated using 300 parallel calculations starting from random initial values to determine the optimal SSR.

To find the global optimum, this process was repeated at least 10 times. As the optimal solution, the flux distribution with the smallest SSR was selected. The goodness of fit was determined *via* a *χ*^2^ test. The 95% confidence intervals were determined based on the Grid-Search procedure published by Antoniewicz et al. [105]. The flux precision of the flux values was determined using the following formula.

$${{{\rm{flux}}}}\,{{{\rm{precision}}}}=\left({{{{\rm{flux}}}}}\,_{{{{\rm{upper}}}}\,{{{\rm{bound}}}}\,{{{\rm{CI}}}}\,95 \% }-{{{{\rm{flux}}}}}\,_{{{{\rm{lower}}}}\,{{{\rm{bound}}}}\,{{{\rm{CI}}}}\,95 \% }\right)/3.92$$

In Table S1 the experimentally measured extracellular rates and mass isotopomer distributions can be viewed.

### Transcriptome sequencing (RNA Seq) and data analysis

0.5 Mio cells were seeded in a six-well plate and incubated for 24 h. The High Pure RNA Isolation Kit (#11828665001, Roche) was used to isolate the total RNA. RNA with an A260/280 ratio >2.0 was used in the experiment and quantified using a NanoDrop (Thermo Scientific). WT and KO cells were analyzed in quadruplicate each.

2500 ng of total RNA were used for Poly-A-RNA library preparation using the D-Plex mRNA-seq Kit (#C05030033, Diagenode), with 12 PCR cycles for library amplification and performing library purification according to the manufacturer’s instructions.

Quality control of libraries was performed using the Qubit™ 1× dsDNA Assay-kit (#Q33230, Thermo Fisher Scientific) regarding library concentration and the Bioanalyzer High Sensitivity DNA Kit (#5037-4626, Agilent Inc.) regarding fragment size analysis.

Libraries were sequenced using the S1 Flowcell on a NovaSeq 6000 (Illumina, San Diego, USA) using 100 bp single-read sequencing.

After sequencing, the quality of reads was analyzed using the FastQC (https://www.bioinformatics.babraham.ac.uk/projects/fastqc/) tool and trimming of adapter-sequences as well as low-quality bases was performed according to the D-Plex mRNA-Seq Kit Manual using the cutadapt tool [106].

Trimmed reads were mapped onto the Human reference genome (Version hg38) by using the TopHat mapper [107] plugin (V7.2.1) for Geneious Prime 2023.1.1. Expression values of annotated genes were calculated using the “calculate expression values” function of Geneious Prime 2023.1.1. and DEGs were analyzed using the Geneious Prime plugin for DESeq2 [108].

The cutoff criteria for the DEGs were a Benjamini and Hochberg adjusted *p* value ≤ 0.01 (BH-adjusted *p* value) and a log2 fold change greater than +1.0 and <−1.0. A complete list of the DEGs of the KO compared to the WT can be found in Table S3. For the DEGs that meet the criteria of BH-adjusted *p* value ≤ 0.001 and FC ≤ -1.3, an enrichment analysis was carried out using the webtool Shiny GO 0.77 with an adjusted p value of 0.05 as threshold criteria, using the false discovery rate method [109] and choosing the Kyoto Encyclopedia of Genes and Genomes database [110] for biological pathways (see Fig. S3).

All sequencing data are available in the ArrayExpress database (http://www.ebi.ac.uk/arrayexpress) under accession number E-MTAB-14271.

### Quantitative real-time reverse transcriptase polymerase chain reaction (qPCR) analysis

qPCR experiments were conducted as previously published [5]. Data were normalized to three reference genes and are shown relative to the control. 18S, HPRT1, and GAPDH were selected as reference genes because all three were stably expressed under the experimental conditions. Gene stability was calculated and found to be *M* < 0.5 for the three mentioned reference genes [111]. Primer sequences are listed in Table S5.

### Lipidomic analysis

0.5 Mio cells were seeded in a six-well plate and incubated for 48 h. Cells were then lysed with 0.2% SDS, transferred to a new tube, diluted with the same volume of water and stored at 80 °C. The protein concentration was determined using a Pierce™ BCA Protein Assay Kit (#23227, Thermo Scientific). Lipidomic analysis was performed in sextuplicate.

For quantitative lipidomics, internal standards were added prior to lipid extraction. An amount of 100 µg protein was subjected to lipid extraction according to the protocol by Bligh and Dyer [112].

The analysis of lipids was performed by direct flow injection analysis (FIA) using a triple quadrupole mass spectrometer (FIA-MS/MS) and a high-resolution hybrid quadrupole-Orbitrap mass spectrometer (FIA-FTMS). FIA-MS/MS was performed in positive ion mode using the analytical setup and strategy described previously [113, 114]. A fragment ion of *m/z* 184 was used for lysophosphatidylcholines (LPC) [114]. The following neutral losses were applied: Phosphatidylethanolamine (PE) and lysophosphatidylethanolamine (LPE) 141, phosphatidylserine (PS) 185, phosphatidylglycerol (PG) 189 and phosphatidylinositol (PI) 277 [115]. Sphingosine-based ceramides (Cer) and hexosylceramides (Hex-Cer) were analyzed using a fragment ion of *m/z* 264 [116]. A detailed description of the FIA-FTMS method was published [117, 118]. Triglycerides (TG), diglycerides (DG) and cholesterol esters (CE) were recorded in positive ion mode as [M + NH_4_]^+^ at a target resolution of 140,000 (at 200 *m/z*). CE species were corrected for their species-specific response [119]. Phosphatidylcholines (PC) and sphingomyelins (SM) were analyzed in negative ion mode as [M + HCOO]^−^ at the same resolution setting. Multiplexed acquisition was applied for the determination of free cholesterol (FC) and the respective internal standard (FC[D7]) [119].

Lipid species were annotated according to the latest proposal for shorthand notation of lipid structures that are derived from mass spectrometry [120]. For FIA-MS/MS glycerophospholipid species, annotation was based on the assumption of even-numbered carbon chains only.

### Luciferase reporter assay

The LDLR plasmid luciferase construct pLDLR-Luc (aka: pES7) was a gift from Axel Nohturfft (Addgene plasmid #14940). The plasmid comprises the sterol regulatory element (SRE) sequence (ATCACCCCAC) for binding of SREBP [121]. 20,000 cells were seeded into wells of a 96-well plate and cotransfected after 24 h. Transfection was conducted with 0.15 µl Lipofectamine 3000 (#L3000015, Thermo Fisher) per well according to the manufacturer’s instructions. 100 ng of each plasmid DNA, a pcDNA3.1 vector, in which the sequence for β-galactosidase was cloned, as a control and the pLDLR-Luc plasmid was used. Luciferase activity was analyzed after 24 h using the Luciferase Assay System (#E4030, Promega) and β-Galactosidase Enzyme Assay System (#E2000, Promega) according to the manufacturer’s instructions with a FLUOstar OPTIMA plate reader (#413-0505, BMG Labtech). Luciferase activity was normalized to β-galactosidase activity to standardize transfection efficiencies.

### Lipid staining

3000 cells per well were seeded in a 96-well plate. For staining of phospholipids, the HCS LipidTOX™ Red phospholipidosis detection reagent (#H34351, Invitrogen) was added 24 h after seeding and incubated for 48 h. Before monitoring, the cells were fixed with 4% paraformaldehyde in PBS for 30 min. For the neutral lipid stain, the cells were incubated for 72 h after seeding, fixed for 30min and afterward stained for 30 min with HCS LipidTOX™ Green neutral lipid stain (#H34475, Invitrogen). After washing with phosphate-buffered saline (PBS), both stainings were analyzed. The images were taken at 10× magnification using the Incucyte® S3 system (Sartorius), with acquisition times of 400 ms for red fluorescence and 300ms for green fluorescence. The image average of the objects' mean fluorescence intensity was calculated using the cell-by-cell mode of the IncuCyte 2020B Software.

### Mitochondria isolation

Functional mitochondria were isolated with the Mitochondria Isolation Kit for Cell Cultures (Thermo Fisher Scientific), supplemented with Halt Protease Inhibitor Cocktail, EDTA-free (Thermo Fisher Scientific), according to the manufacturer’s instructions.

### Citrate measurement by gas chromatography-mass spectrometry (GC-MS)

The pellets of isolated mitochondria were carefully resuspended in 200 µl of the respective mitochondrial buffer (sucrose 70 mM, mannitol 220 mM, KH_2_PO_4_ 10 mM, magnesium chloride 5 mM, 2-(4-(2-hydroxyethyl)-1-piperazinyl)-ethanesulfonic acid 2 mM, ethylene glycol-bis(β-aminoethyl ether)-*N,N,N’,N’*-tetraacetic acid 1 mM, fatty acid free BSA 0.2% (m/V), nicotinamide adenine dinucleotide (NADH) 0.2 mM, U¹³C-labeled glutamine 4 mM, pyruvate 0.2 mM) and incubated for 1 h at 37 °C and 5% CO_2_. After incubation, the preparations were centrifuged for 10 min at 8000 × *g* and 37 °C. The supernatant was removed, resuspended with 1 ml PBS and centrifuged for 5 minutes at 8000 × *g* at 37 °C. The supernatant was removed, and the mitochondrial pellet was resuspended with 60 µl ice-cold methanol and vortexed. Between 8 and 10 preparations per genotype were combined, resulting in 460–600 µl of mitochondrial suspension, quenched in methanol, and stored at −80 °C until GC-MS measurement. The mass isotopomer distribution of citrate was measured using an Agilent 7890B/7200 GC/quadrupole time of flight with an EI source. For this purpose, 600 µl mitochondria-methanol mixture was completely dried in a 1 ml conical glass vial using SpeedVac. The dried sample was dissolved in 15 µl methoxiamine (1 mg/ml in pyridine) and shaken at 60 °C for 60 min. 30 µl N-tert-Butyldimethylsilyl-N-methyltrifluoroacetamide (MBDSTFA) was added and shaken at 60 °C for 60 min. The derivatized sample was transferred to a glass insert. 1 µl of the derivatized sample was injected. The helium flow was 1 ml/min, the EI source operated at 70 eV and 230 °C, and the liner and transfer line were at 250 °C. The oven program consisted of 60 °C for 1 min followed by a ramp of 10 °C/minute up to 325 °C, which was held for 10 min. The solvent delay was 4.7 minutes. Raw data were analyzed using the MassHunter Quantitative Analysis Software (Agilent, B.10.00, Santa Clara, USA).

### CLIP data analysis

Publicly available enhanced CLIP (eCLIP) RNA binding data from the ENCODE consortium for HepG2 liver cancer cells and K562 cells were obtained and analyzed as previously described [5, 122]. eCLIP peaks were obtained from the ENCODE data portal (https://www.encodeproject.org/). Each annotated human gene in the Ensembl database that had an eCLIP peak in at least one of the two cell lines was denoted as an IMP2 target gene.

### Statistical analysis

Data analysis and statistics were performed using Origin Pro version 19 software. Data are shown as mean values ± SD unless otherwise stated. Depending on the normality of the data, as assessed by the Shapiro-Wilk Test, statistical differences were calculated using a two-sided Student’s *t* test or a Mann–Whitney *U* Test. For multiple comparisons, a two-way ANOVA was performed. Biological replicates were represented as “*n*” and technical replicates (wells per experiment) were represented as indicated numbers in brackets behind them. *p ≤ 0.05, **p ≤ 0.01, ***p ≤ 0.001.

## Supplementary information


Overview Supplementary Material
Supplementary figure S1–S6
Supplementary table S1
Supplementary table S2
Supplementary table S3
Supplementary table S4-S5


## Data Availability

Further information and requests for resources and reagents should be directed to and will be fulfilled by the lead contact, Sonja M. Kessler (pharmakologie@phamazie.uni-halle.de). All sequencing data are available in the ArrayExpress database (http://www.ebi.ac.uk/arrayexpress) under accession number E-MTAB-14271.
