# Supplementary material for: The insulin-like growth factor 2 mRNA-binding protein 2 affects tumor cell metabolism via mitochondrial transporter activity and lipid alterations
Source: Cell Death Discov. 2026 Sep 1;12:353. doi: 10.1038/s41420-026-03315-4 (PMC13534510; doi:10.1038/s41420-026-03315-4)
Supplement: Supplementary file 1 [file 41420_2026_3315_MOESM1_ESM.pdf]

Supplementary Materials

Supplementary file S1: LINEX network.

Figure S1: Metabolic flux maps of HCT116 WT and HCT116 IGF2BP2 KO cells

Figure S2: Principal component analysis.

Figure S3: Enrichment analysis of differentially expressed genes (DEGs) of HCT116 IGF2BP2 knockout cell line.

Figure S4. Uncropped Western blot 1 related to figure 4.

Figure S5. Uncropped Western blot 2 related to figure 5

Figure S6: Confirmation of HCT116 IGF2BP2 knockout by western blot analysis.

Table S1: 13C-metabolic flux analysis.

Table S2: Lipidome and FAME raw data.

Table S3: Transcriptome sequencing.

Table S4: List of possible targets of IGF2BP2 in RNA-Seq compared to qPCR and publicly available CLIP and RIP data.

Table S5: Primer sequences
