# Supplementary material for: The insulin-like growth factor 2 mRNA-binding protein 2 affects tumor cell metabolism via mitochondrial transporter activity and lipid alterations
Source: Cell Death Discov. 2026 Sep 1;12:353. doi: 10.1038/s41420-026-03315-4 (PMC13534510; doi:10.1038/s41420-026-03315-4)
Supplement: Supplementary file 2 [file 41420_2026_3315_MOESM6_ESM.pdf]

Supplementary Tables S4 and S5

Table S4: List of possible targets of IGF2BP2 in RNA-Seq compared to qPCR and publicly available CLIP [1, 2] and RIP data [3]. RNA-Seq data (see table S3) and qPCR data (see Fig. 3) of the HCT116 wildtype in contrast to the IGF2BP2 knockout cell line were compared to the publicly available CLIP and RIP data. RNA-Seq and qPCR data are shown as significant up (↑) and down (↓) regulation, whereas a checkmark for CLIP and RIP data shows the corresponding IGF2BP2 binding effect. (<>) Stands for not significant**.**

gene_name	genesis	RNA Seq Down (↓)-/Upregulation (↑)	qPCR Down(↓)-/Upregulation (↑)	CLIP	RIP	Differential Expression	
						Adjusted *p*-value	Log2 Ratio
ACACA	ENSG00000278540	<>		✓	✓	0.090	-0.173
ACLY	ENSG00000131473	<>		✓	✓	0.792	-0.040
CPT1A	ENSG00000110090	↓		✓		1.1E-06	-0.450
ELOVL2	ENSG00000197977	<>				0.949	0.025
ELOVL3	ENSG00000119915	↑				0.010	0.509
ELOVL5	ENSG00000012660	<>		✓	✓	0.383	0.086
ELOVL6	ENSG00000170522	<>	↓	✓		0.998	-3.8 E-04
ELOVL7	ENSG00000164181	↑		✓		0.015	0.238
FADS2	ENSG00000134824	<>		✓	✓	0.404	-0.067
FASN	ENSG00000169710	↓	↓	✓	✓	2.5E-08	-0.363
SLC2A12 (Glut1)	ENSG00000117394	<>		✓	✓	0.082	0.374
GTP2	ENSG00000166123	↓		✓	✓	0.002	-0.351
HMGCR	ENSG00000113161	<>	↑	✓	✓	0.675	0.086
IDH2	ENSG00000182054	↑	↑	✓		1.2E-04	0.440
IDH3A	ENSG00000166411	↓	<>	✓	✓	0.005	-0.246
LDLR	ENSG00000130164	↑		✓		4.9E-08	0.460
MPC1	ENSG00000060762	↓	↓	✓		0.001	-0.342
MYC	ENSG00000136997	↓		✓	✓	2.3E-14	-0.771
PC	ENSG00000173599	↑	↑	✓		0.014	0.340
PPARA	ENSG00000186951	↑	↑	✓		2.0E-05	0.424
SCAP	ENSG00000114650	<>		✓	✓	0.421	-0.091
SCD	ENSG00000099194	↑	↑	✓	✓	4.3E-17	0.509
SDHD	ENSG00000204370	↓	↓	✓		2.4E-05	-0.419
SLC1A5	ENSG00000105281	↓		✓	✓	0.001	-0.254
SLC25A10	ENSG00000183048	↑	↑	✓		1.1E-05	0.556

SLC27A1	ENSG00000130304	↑				1.0E-06	0.611
SLC27A2	ENSG00000140284	↑		✓		2.0E-06	0.423
SLC27A3	ENSG00000143554	<>				0.149	0.271
SLC27A4	ENSG00000167114	<>		✓		0.677	0.053
SLC27A5	ENSG00000083807	<>		✓		0.233	-0.200
SLC27A6	ENSG00000113396	↑				2.9E-10	0.831
SREBF1	ENSG00000072310	↑		✓	✓	1.1E-08	0.396
SREBF2	ENSG00000198911	<>		✓	✓	0.155	0.203
UCP2	ENSG00000175567	↑	↑	✓	✓	7.9E-14	0.969

[1] Kendzia, S.; Franke, S.; Krohler, T.; Golob-Schwarzl, N.; Schweiger, C.; Toeglhofer, A. M.; Skofler, C.; Uranitsch, S.; El-Heliebi, A.; Fuchs, J.; et al. A combined computational and functional approach identifies IGF2BP2 as a driver of chemoresistance in a wide array of pre-clinical models of colorectal cancer. *Mol Cancer* 2023, *22* (1), 89. DOI: 10.1186/s12943-023-01787-x From NLM Medline.
[2] Dehghani Amirabad, A.; Ramasamy, P.; Wierz, M.; Nordstrom, K.; Kessler, S. M.; Schulz, M. H.; Simon, M. Transgenic expression of the RNA binding protein IMP2 stabilizes miRNA targets in murine microsteatosis. *Biochim Biophys Acta Mol Basis Dis* 2018, *1864* (10), 3099-3108. DOI: 10.1016/j.bbadis.2018.05.024 From NLM Medline.
[3] Huang H, Weng H, Sun W, Qin X, Shi H, Wu H, et al. Recognition of RNA N(6)-methyladenosine by IGF2BP proteins enhances mRNA stability and translation. *Nat Cell Biol.* 2018;20(3):285-95

Table S5: Primer sequences.

gene	Primer Forward (5´-3´)	Primer Reward (5´-3´)	Ref.
Housekeeping genes			
18S	AGGTCTGTGATGCCCTTAGA	GAATGGGGTTCAACGGGTTA	
ACTB	TGCGTGACATTAAGGAGAAG	GTCAGGCAGCTCGTAGCTCT	
GAPDH	GTCTCCTCTGACTTCAACAG CG	ACCACCCTGTTGCTGTAGCC AA	
HPRT1	TGACCTTGATTTATTTTGCAT ACC	TGAGGAATAAACACCCTTTC CA	
IGF2BP2			
IGF2BP2	GTTCCCGCATCATCACTCTTA T	GAATCTCGCCAGCTGTTTGA	
Primer sequences related to genes of the mitochondrial metabolism			
SLC25A10	CTTAGCACCGGGTACCTCTC	GCACATCCACCTGCAATAAA	4
UCP2	GTCCGGTTACAGATCCAA GGAG	AGCCCATTGTAGAGGCTT CG	5
MPC1	ACCACTCGGGGTCTGGTGT	GGAAATCCTTGCTTCGGACA	
IDH2	CGGACTTCCTCGACACCATC	CCCCTAGAAAGGCCTCCAGA	
IDH3A	GCCCTTTGAAGACCCCAATA	GTCGGACATTCGCGTAAAGG	
PC	TCCCCAAGCCTTCAACAGAA	GAGGAAAGGACGATGGCTG A	
SDHD	CTCCAAGGCTGCATCTCTCC	GTCCATCGCAGAGCAAGGAT	
Primer sequences related to genes of the lipid metabolism			
ELOVL6	CACTGTGAGCTGGAAAAGGG AG	GTGTGAAGTCAAACAGGGAG GG	
FASN	ACTTCCCCAACGGTTCAGGT TC	GCGTCTTCCACACTATGCTC AG	
SCD	GCCAATTTCCTCTCCACTGC TG	AAGTTCGCTCTTAGAAGCTG CC	
HMGCR	GCAGGACCCCTTTGCTTAGA	GCACCTCCACCAAGACCTAT	
PPARA	TGCGTAGAAGAGCCCAGAAA	GTTGACTGGACGGAGCTGAG	

[4] Zhou X PJ, Krishnan S, Curbo S, Karlsson A. The mitochondrial carrier SLC25A10 regulates cancer cell growth. *Oncotarget*. 2015;6(11):9271-83.
[5] Segales J, Sanchez-Martin C, Pujol-Morcillo A, Martin-Ruiz M, de Los Santos P, Lobato-Alonso D, et al. Role of UCP2 in the Energy Metabolism of the Cancer Cell Line A549. *Int J Mol Sci.* 2023;24(9).
